# Effectiveness of different types of mental simulation in the weight loss process based on a perseverance study among people with different BMI

**DOI:** 10.1186/s13690-021-00524-4

**Published:** 2021-01-19

**Authors:** Wojciech Styk, Szymon Zmorzyński, Waldemar Klinkosz

**Affiliations:** 1 Institute of Psychology, The John Paul II Catholic University of Lublin, Al. Raclawickie 14, 20-950 Lublin, Poland; 2 Department of Cancer Genetics with Cytogenetic Laboratory, Medical University of Lublin, Al. Raclawickie 1, Lublin, 20-059 Poland; 3 Institute of Psychology, Cardinal Stefan Wyszynski University in Warsaw, Dewajtis 5, Warsaw, 01-815 Poland

**Keywords:** Mental imagery, Mental simulations, Perseverance, BMI, Obesity, Overweight, Resistance to distractors

## Abstract

**Background:**

Most of the world’s population lives in countries in which overweight and obesity kill more people than does underweight. The weight loss process can be supported by mental simulations, which are used to help individuals to effectively strive towards various goals. The aim of this study was to determine the impact of different types of mental simulations on perseverance, resistance to distractors and the ability to inhibit irrelevant thoughts or memories in people with different body mass indexes (BMI).

**Methods:**

The study included 252 participants. They performed process simulations and outcome simulations, using instructions presented to them during the experiment. Perseverance and resistance to distractors were determined using a computer maze-solving task. Two indicators of perseverance were analysed: number of maze tasks solved and total time spent on solving the test. Mean time spent on a single task was used as a measure of resistance to distractors and the ability to inhibit irrelevant thoughts and memories.

**Results:**

The results of the analyses showed that the type of mental simulation used had an effect on the indicators of perseverance. Process simulation subjects completed more tasks and spent more time solving the test than outcome simulation subjects. A relationship was found between the subjects’ BMI and the investigated indicators. Individuals who were underweight, overweight or obese scored lower on all three indicators compared to subjects with normal BMI. In people with a BMI above normal, mental simulations increased resistance to distractors and the ability to inhibit thoughts sidetracking them from the task at hand. It is possible that increasing the resistance to distractors is responsible for the effectiveness of mental simulations in the weight loss process.

**Conclusion:**

Our results can be applied in developing interventions for people who suffer from overweight and obesity. Psychological interventions based on mental simulations can be used to assist individuals in physical activity, leading to an improvement in health, but it has to be underlined that the mechanism of their action may vary from person to person.

## Background

### Overweight and obesity

Overweight and obesity have become global concerns. The incidence of obesity across the world has almost tripled since 1975. Most of the world’s population lives in countries in which overweight and obesity kill more people than does underweight. Abnormally high body weight may lead to chronic illnesses such as cardiovascular diseases, diabetes and certain cancers [1]. It may also be associated with low self-esteem [2]. In recent years, a large number of studies have investigated the problem of comorbidity of depression and depressed mood symptoms with obesity and overweight [3–5]. Given the prevalence and seriousness of the phenomenon, scientists have been focusing on developing methods that can help people suffering from obesity and overweight to lose weight. Overweight is caused by an imbalance between the amount of energy taken as food and the amount of energy used by the body [6]. Creating a negative energy balance is the only way to reduce body weight. This can be achieved by minimizing the amount of energy taken in (the number of calories consumed) or by increasing energy spent, e.g. through physical activity. Contemporary studies indicate that the most effective weight loss strategies include a diet with a reduced calorie intake and increased physical activity [7, 8]. However, each of the proposed strategies requires patients to persist in their actions. Some studies show, that obese and overweight people have difficulty inhibiting irrelevant thoughts or memories and that this may result in poorer perseverance [9, 10]. Persistence in dieting and exercising in order to lose weight is part of a wider psychological problem of effective self-control and self-regulation [11].

An important research topic related to overweight and obesity is the relationship between body image and perseverance in action. In a study by Styk et al., correlations were found between the subjects’ body image and their perseverance [10]. People who perceived their body weight as being too high had lower scores on perseverance indicators. The effect size of this relationship was moderate in the group of subjects with normal BMI and large in the group of subjects with high BMI. Styk et al. also observed a positive correlation between BMI and the indicator describing concentration and resistance to distractors. Dissatisfaction with one’s body image is related to weight self-stigma [12]. A negative body image can be associated with poor psychological functioning, e.g. low perseverance. Studies of overweight and obese people show that they are characterized by a lower self-esteem and a lower self-efficacy [12, 13]. Social discrimination and stereotypes about overweight and obese people may lead them to believe that they are lazy and impersistent [12, 14, 15]. Weaker perseverance in the group of obese patients may be related to these stereotypes. Once internalized, they can result in weight-related stigma and devaluation of self-esteem.

### Mental simulations in the goal achievement process

As shown by previous research, actions performed on imaginary objects share much in common with real-life actions [16, 17]. In a study by Ranganathan et al., muscle strength gains were compared between a motor imagery training group and a physical training group. The physical training group increased muscle strength by 53%, while the imagery training group saw a 35% increase in muscle strength. There were no significant changes in the control group [18]. A fMRI study has shown that the image of a face and its actual perception have common processing mechanisms. That study has also demonstrated that the content of the visual image determines what brain regions are activated during mental imaging [19].

Mental simulations are a special form of mental imagery. They are defined as imitative representations of an event or a series of events [20]. They can be divided into three basic types [21]: outcome simulations, i.e. representations of the final effect of an action; process simulations, i.e. representations of an action plan leading to the achievement of an objective; and meditations, i.e. negative images of failure and anxiety. Research suggests that process simulations may facilitate effective goal-orientation, while outcome simulations and meditations interfere with self-regulation [22–25].

Zimmerman and Kitsantas have demonstrated that when people are learning to cope with a new task, focusing on the process helps them obtain the means essential to achieving the goal [26]. In contrast, concentrating on the outcome diverts their attention from acquiring and practising the use of the means that are necessary to reach the objective, thus hindering the successful pursuit of the goal. In a study which confirmed the potential negative impact of focusing on the outcome on pursuing the difficult goal of losing weight, Oettingen and Wadden showed that positive weight loss fantasies were negatively correlated with actual weight loss [27]. According to those authors, indulging in positive fantasies may have inclined dieters to dream of positive outcomes without having to engage in the more difficult pursuit of their dietary goal. This and other studies conducted by Oettingen suggest that concentrating on positive outcomes is rather detrimental. Focusing on the final result can distract people from taking measures related to the achievement of their goal and therefore can make it difficult to achieve the goal [22, 23]. Especially when pursuing long-term objectives, focusing on the means to the goal rather than the negative discrepancy between the current state and the desired end-state should keep one motivated even in the face of obstacles or setbacks [28].

Research on cognitive behavioural therapy also illustrates the importance of mental simulations in achieving goals. In particular, Marlatt et al.’s relapse prevention techniques show how important mental simulation of a high-risk relapse situation can be in helping people abstain from health-threatening behaviours such as smoking and excessive drinking [29]. For example, a person trying to overcome a drinking problem can practice mentally how they are going to refrain from drinking alcohol during a meeting with friends. By doing so, they can develop and refine the specific coping skills they will need to avoid temptations diverting them from achieving their goal [29, 30].

The results of research by Marszal-Wisniewska and Jarczewska-Gerc [24, 31] on the role of mental simulations in the weight loss process clearly show that imagining the process of weight loss increases the effectiveness of action. The authoresses show that mental simulations affect the respondents’ perseverance, thus increasing the efficiency of the weight loss process.

### The aim of this study

As the literature shows, there are many studies investigating the influence of mental simulations on perseverance and goal achievement, but still some questions remain unanswered. Do mental simulations affect subjects’ perseverance by strengthening their self-control, or do they rather allow individuals to focus more on achieving a goal as they increase their resistance to distractions diverting their attention away from the goal?

The relationship between the effectiveness of different types of mental simulations and the achievement of normal body mass has not been studied yet. Given this research gap, we wanted to determine the effect of two different types of mental simulations on perseverance in action in people with different BMI, and the relationship of the type of mental simulation with focusing on a goal and resistance to distractors.

## Materials and methods

### Participants

The study included 252 people, 56% of females (*N*=141) and 44% of males (*N*=111). The youngest subject was 18 years old, and the oldest was 58 years old. The mean age was 35 years (SD=11). The subjects had their BMI measured: 20 subjects (7%) had a body weight below normal, 160 subjects (65%) had a normal body weight, and 72 (28%) had a body weight above normal. Most of the subjects had a higher education degree (82%).

### Procedure

The study was conducted using a website. Participants were recruited through on-line forums, e-mail invitations, and social networking sites. The recruitment sites and modes were not related to the problems of overweight and weight loss. Mailing lists were obtained from an address base collected from individuals who had given their consent to participate in a different study. The snowball sampling method was used, which means the subjects were asked to invite other persons to take part in the experiment. The study did not place any restrictions on the number of subjects. Only those individuals who gave their consent to participating in the study were enrolled and could proceed to the actual test. The website also contained a note about the aim of the study and a description of the test procedure. The study was divided into two stages. In the first stage, the participants completed a demographic survey with questions regarding their gender, age, education, body weight and height. In the second stage, the subjects were randomly assigned either to an outcome mental simulation group or a process mental simulation group.

The subjects received instructions how to run the simulation and were presented with the Maze Test [10]. Perseverance was measured several times by determining various indicators of perseverance during the performance of repetitive tasks [25, 32, 33]. In these experiments, perseverance was understood as the ability to overcome discomfort resulting from the desire to give up, the ability to overcome obstacles such as distractors and uncontrolled distractive thoughts, and the ability to control the desire to engage in substitute activities. In the Maze Test, easy maze tasks were used, so as to reduce the discomfort that the difficulty of solving the test might produce in the subjects, and to minimize interruptions caused by the subjects’ not being able to solve a task.

**Fig. 1:**
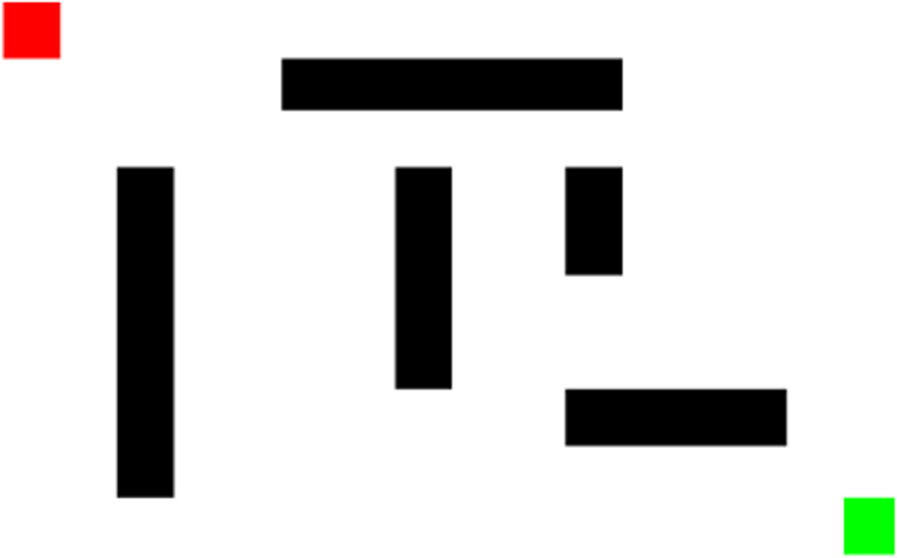
An example of a maze task

The subjects were asked to solve as many maze tasks as possible and were not given a time limit. To solve a maze, the participants had to move the mouse cursor from a green point to a red point without crossing the inner “walls” of the maze. A sample maze is shown in Fig. 1.

Next, the subjects were assigned to the outcome and process simulation groups alternately to ensure that the groups were as equal in size as possible. The subjects were assigned to the groups randomly, i.e. independent of any particular variables.

The number of attempts was unlimited, as it was important that the subjects achieved the goal, i.e. solved a given maze task. Before attempting the test, each participant could solve one trial maze. Once, a person passed through a maze, another randomly allocated maze was displayed on the computer screen. The subjects could quit the test at any time by clicking the “Quit” button.

The Maze Test permits to determine two perseverance indicators: (1) the number of maze tasks solved, which defines the subjects’ perseverance taking into account task solving efficiency, and (2) the total time spent on solving the test – an indicator which does not take into account the efficiency of task solving, but merely the time it takes a person to solve the test.

Additionally the Maze Test also allows to determine the mean time spent on a single task, which quantifies a subject’s resistance to distractors and ability to inhibit irrelevant thoughts or memories. This indicator was calculated as a quotient of the total time spent on the whole test and the number of tasks solved. If the subjects are not sufficiently focused on the task and get involved in activities or thoughts that distract them from the task being solved, the value of this indicator will be high. Low values of this indicator will be obtained when the subjects are able to inhibit irrelevant thoughts and resist distractors, i.e. when they solve the test without being distracted from it [10]. The mean time spent on one task is only an indicator of focus, which may or may not be related to perseverance. A subject who solves 10 tasks in 10 s will have a score of 1 on this indicator, similarly to a subject who solves 200 tasks in 200 s. However, the perseverance indicators of these subjects will be different.

### Mental simulations

Before the test was started, the participants had to run a simulation and read the instruction for their group (outcome vs. process). The instruction for the outcome simulation focused the subjects’ attention on the results of their work and contained the following phrases: “imagine that you get the best result on this task”, “focus on the result”, “think only about the result”, “imagine that you have eventually achieved your goal – you are the best”, “your friends will congratulate you on your result and will be impressed you have achieved such a high score”. The process simulation instruction focused the subjects’ attention on the process of solving the task and contained the following phrases: “imagine that you walk through the maze”, “try to imagine how you solve it”, “imagine every move one by one”, “think whether there is a better solution”.

### Body mass index

The subjects’ nutritional status was assessed using the BMI, calculated as weight in kilograms divided by height in meters squared. The World Health Organization (WHO) defines overweight adults as those who have a BMI of 25–29.9 kg/m2; obesity is defined as BMI ≥ 30.0 kg/m2. The normal BMI range is 18.5–24.9 kg/m2. Individuals with a BMI below 18.5 kg/m2 are considered underweight [34]. In the present study, the BMI was calculated based on the weight and height values declared by the subjects.

### Data analysis

When designing the study, we determined that the number of respondents should be at least 200 people to obtain a test with a sufficient power. This condition was met in the present experiments [35]. Data analysis showed that all the investigated variables had a normal distribution. The dependent variables did not take values below or above three standard deviations. Thus, no corrections were made. The groups did not differ significantly in the levels of the variables, which suggests that the randomisation produced balanced groups. Because gender, age and education did not differentiate statistically between the groups, these variables were not included in the analysis.

The data were assessed statistically using ANOVA variance analysis and Student’s t-test. A 2×3ANOVA was performed (two types of mental simulations and three BMI groups: below normal, normal and above normal). The calculations were made using Statistica software version 13.

## Results

### Number of tasks solved – a perseverance indicator that takes into account the efficiency of action

**Fig. 2:**
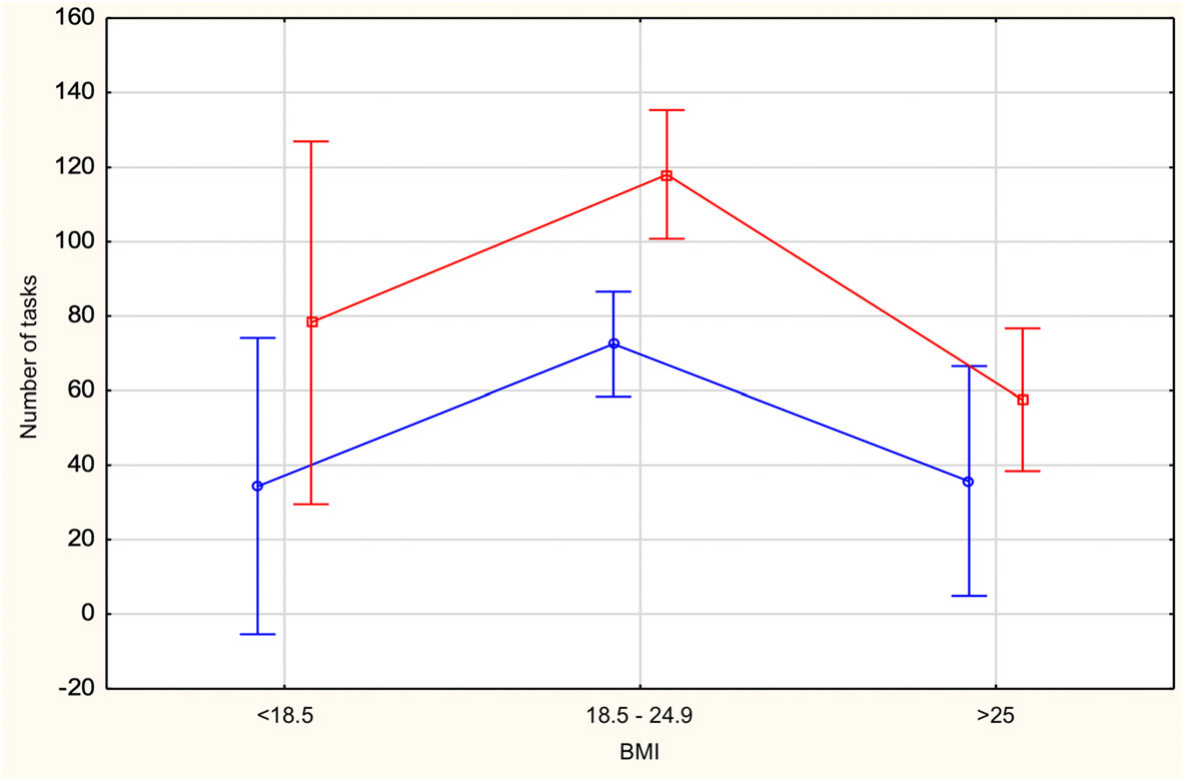
Number of tasks solved by subjects with below-normal, normal and above-normal body weight in the online Maze Test, 2019. Process simulations are marked in red and outcome simulations are marked in blue

The effect of interaction of the main factors was nonsignificant. The subjects’ results by BMI group are shown in Fig. 2.

The main effect of type of mental simulation was significant F (1)=8.32; *p*< 0.001; eta2=0.03. The type of mental simulation used affected the number of tasks solved. Process simulation subjects completed, on average, more tasks (M = 90.10; SD = 89.47) than outcome simulation subjects (M = 63.17; SD = 53.27). The significance of the difference was p<.001 The results showing the effect of the type of mental simulation on the investigated perseverance indicators are given in Table 1.

**Table 1:** Effect of type of simulation on perseverance indicators in the online Maze Test, 2019

	number of completed tasks	total time spent on tasks [s]
	*M (SD)*	*M (SD)*
Process simulation	90.10 (89.47)	687.43 (611.18)
Outcome simulation	63.17 (53.27)	473.89 (361.46)

The main effect of BMI group also turned out to be significant F (2)=11.33; *p*< 0.01; eta2=0.08. It was stronger than the main effect of type of mental simulation. This means that BMI group differentiated the number of tasks solved by the subjects. Normal BMI subjects solved, on average, more tasks (M = 90.72; SD = 83.54) than subjects with BMI below normal (M = 51.90; SD = 47.30) and subjects with BMI above normal (M = 51.47; SD = 45.73). The significance of the difference was *p* < 0.001. There were no significant differences between the underweight group and the overweight/obese group. The results are shown in Table 2.

**Table 2:** Effect of BMI on perseverance indicators in the online Maze Test, 2019

	number of completed tasks	total time spent on tasks [s]	mean time spent on one task [s]
	*M (SD)*	*M (SD)*	*M (SD)*
BMI < 18.5	51.90 (47.30)	396.15 (216.12)	10.97 (6.08)
18.5 ≤ BMI ≤ 24.9	90.72 (83.54)	666.04 (566.49)	8.25 (2.93)
BMI > 25	51.47 (45.73)	436.25 (378.09)	11.74 (9.29)

### The total time spent on tasks – an indicator that does not take into account the efficiency of solving the tasks

**Fig. 3:**
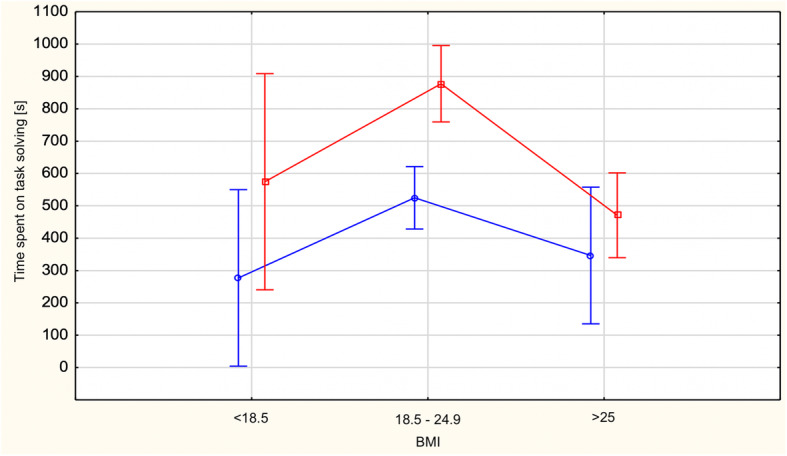
Total time spent on task solving [s] for subjects with below-normal, normal and above-normal body weight in the online Maze Test, 2019. Process simulations are marked in red and outcome simulations are marked in blue

The effect of interaction of the main factors was, again, nonsignificant. The subjects’ scores by BMI are shown in Fig. 3.

The analysis demonstrated the significance of the main effect of type of mental simulation F (1)=8.57; *p*< 0.01; eta2=0.03. The subjects who had run the process simulation spent, on average, significantly more (*p* < 0.01) time on solving the tasks (M = 687.43; SD = 611.18) than the outcome simulation subjects (M = 473.89; SD = 361.46). The significance of the difference was *p*<.001 The effects of mental simulations on the investigated perseverance indicators are given in Table 1.

The main effect of BMI group turned out to be significant F (2)=9.27; *p*< 0.001; eta2=0.07. Like in the case of the number of tasks solved, BMI differentiated the total time spent on solving the test. The main effect of BMI, similarly as in the case of the number of tasks performed, was stronger than the main effect of type of mental simulation. Normal BMI subjects spent, on average, more time on solving the test (M= 666.04; SD=566.49) than the subjects with BMI below normal (M= 396.15; SD=216.12) and the subjects with BMI above normal (M=436.25; SD=378.09). The significance of these both differences was < 0.001. There were no significant differences between the underweight group and the overweight/obese group. The results of the analyses are shown in Table 2.

### Mean time spent on one task – an indicator of resistance to distractors and the ability to inhibit irrelevant thoughts and memories

**Fig. 4:**
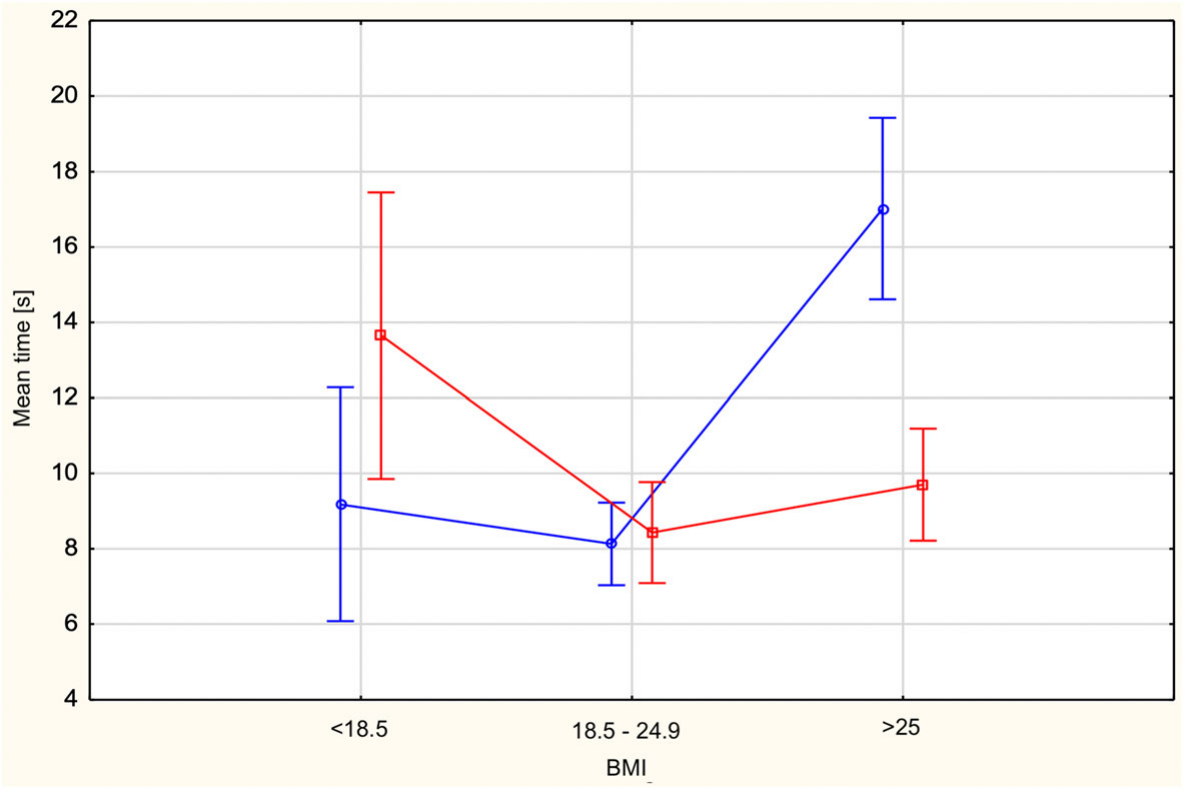
Mean time [s] spent on one task by subjects with below-normal, normal and above-normal body weight in the online Maze Test, 2019. Process simulations are marked in red and outcome simulations are marked in blue

The effect of interaction of the main factors was significant F (2)=13.12; *p*< 0.01; eta2=0.10. The subjects’ results by BMI group are shown in Fig. 4.

The effectiveness of the two types of mental simulation was analysed separately for below normal, normal and above normal BMI groups. Differences between mental simulations (process vs outcome) were significant only in the group of people with a body weight above normal (M_process_=9.7, SD=4.92; M_outcome_=17.02, SD=14.72; *p*< 0.01). This means that the type of mental simulation used influenced the mean time spent on the task only in the group of overweight people. Overweight and obese people who had performed the process simulation spent, on average, significantly less time solving a single task. The process simulation had a greater impact on resistance to distractors and the ability to inhibit insignificant thoughts and memories in the group of overweight and obese people. The results are shown in Table 3.

**Table 3:** Interaction effect for mean time spent on solving one task in the online Maze Test, 2019

	Process simulation	Outcome simulation	t	df	p
	*M (SD)*	*M (SD)*			
BMI < 18.5	13.65 (8.73)	9.18 (2.58)	1.69	18	0.11
18.5 ≤ BMI ≤ 24.9	8.43 (2.38)	8.13 (3.25)	0.64	158	0.52
BMI > 25	9.70 (4,92)	17.02 (14.72)	−3.18	70	< 0.01

The analysis demonstrated that the main effect of type of mental simulation was nonsignificant F (1)=0.71; *p*=0.40. The type of mental simulation the subjects had run did not have a significant effect on the indicator describing resistance to distractors.

The main effect of BMI turned out to be significant F (2)=19.11; *p*< 0.001; eta2=0.13. This effect was the strongest in this study and explained 13% of variance in the mean time spent on one task. Normal BMI subjects needed, on average, less time to solve one task (M= 8.25; SD=2.93) than the subjects with BMI below normal (M= 10.97; SD=6.08) and the subjects with BMI above normal (M=11.74; SD=9.29). The significance of these both differences was *p*< 0.001 There were no significant differences between the underweight group and the overweight/obese group. The results are shown in Table 2.

## Discussion

To the best of our knowledge, the present study is the first one to investigate the influence of mental simulations on perseverance in people with different BMI. We found that the type of mental simulation used affected both the number of tasks solved and the total time spent on solving the test. Individuals who had run the process simulation, achieved higher perseverance indicator values than those who had performed the outcome simulation. This finding is consistent with previous research on mental simulations [25, 36].

Attention should also be paid to the lower values of perseverance indicators in the groups of people with BMI outside the normal range. Both the overweight/obese subjects and the underweight subjects scored significantly lower on all three perseverance indicators. BMI explained as much as 13% of variance in the variable describing the subjects’ concentration. In the group of people with overweight and obesity, this indicator was clearly affected by the type of mental simulation used. The effect of type of mental simulation explained 10% of variance in the variable describing the subjects’ concentration.

Previous research on the relationship between the type of mental simulation used and weight loss showed that people subjected to a process simulation lost significantly more weight than those who performed an outcome simulation [24, 31]. This effect was probably associated with the influence of mental simulations on resistance to distractors and the ability to control irrelevant thoughts, rather than their direct impact on perseverance. Previous studies demonstrate that obese and overweight people have difficulty inhibiting irrelevant thoughts and memories [9]. The use of process simulations in people with a high BMI can improve their resistance to distractors and the ability to inhibit thoughts distracting them from the task at hand.

Perseverance and goal achievement are also undoubtedly related to an individual’s resources and physical and mental health condition [37–39]. A study on depression and depressed mood symptoms in people with different BMI found a U-shaped relationship between these variables [5]. People with low and high BMI are more likely to suffer from those symptoms than individuals with normal BMI. Lower rates in people with abnormal BMI may result from depression-associated factors. This present study did not take into account factors associated with depression and reduced mood. It is advisable to take these factors into account in future studies.

### Clinical implication

The present results can be used to support people struggling with overweight and obesity. In working with overweight and obese patients, health professionals should focus on the process of reaching the goal of losing weight, paying particular attention to the difficulties that may occur along the way. This approach is similar to the use of mental simulations in addiction treatment [30]. Patients should be made aware of the source of the effectiveness of mental simulation therapy and the fact that it can increase their resistance to distracting factors which, when uncontrolled, may prevent them from achieving their goals. In treating overweight and obese patients, any suggestions that successful weight loss depends only on perseverance should be avoided, as they may reinforce the negative stereotypes patients may hold about overweight and obese people being less persistent. These stereotypes are related to the patients’ negative body image, which, in turn, may have a negative impact on the outcomes of their therapy [10, 40].

### Limitations

It should be emphasized that in our study, the weight and height data were self-reported, which means that BMI determination may have been distorted. To eliminate this potential error, independent measurements of height and weight should be made in future studies. Also, when selecting the study group, it would be advisable to exclude people with possible metabolic disorders. Furthermore, the study did not take into account factors associated with depression and depressed mood. To enhance the statistical power of the analysis, future studies can be designed to include random effect models that allow the same people to be tested multiple times.

## Conclusion

The results of the analyses show that the type of mental simulation used has an effect on perseverance indicators. A relationship was also found between the subjects’ BMI and the investigated indicators of perseverance. The analysis of the perseverance indicators showed no interaction between the type of mental simulation used and BMI. In people with a BMI above normal, mental simulations affected their resistance to distractors and the ability to inhibit distracting thoughts. These findings can be used to develop interventions for people who suffer from overweight and obesity. Interventions based on mental simulations can be used to assist patients in physical activity, leading to an improvement in health. It has to be remembered though that the mechanism of action of these interventions may vary from one person to another.

## Data Availability

The datasets used and/or analysed during the current study are available from the corresponding author on reasonable request.

## Abbreviation

BMI: Body Mass Index
